# The impact of workload on hand hygiene compliance: Is 100% compliance achievable?

**DOI:** 10.1017/ice.2021.179

**Published:** 2022-09

**Authors:** Nai-Chung N. Chang, Marin L. Schweizer, Heather Schacht Reisinger, Michael Jones, Elizabeth Chrischilles, Margaret Chorazy, W. Charles Huskins, Loreen Herwaldt

**Affiliations:** 1 Department of Epidemiology, University of Iowa College of Public Health, Iowa City, Iowa; 2 Division of Epidemiology, University of Utah School of Medicine, Salt Lake City, Utah; 3 Veterans’ Affairs Salt Lake City Health Care System, Salt Lake City, Utah; 4 Iowa City Veterans’ Affairs Health Care System, Iowa City, Iowa; 5 Department of Internal Medicine, University of Iowa Carver College of Medicine, Iowa City, Iowa; 6 Department of Biostatistics, University of Iowa College of Public Health, Iowa City, Iowa; 7 Mayo Clinic College of Medicine and Science, Rochester, Minnesota

## Abstract

Hand hygiene compliance decreased significantly when opportunities exceeded 30 per hour. At higher workloads, the number of healthcare worker types involved and the proportion of hand hygiene opportunities for which physicians and other healthcare workers were responsible increased. Thus, care complexity and risk to patients may both increase with workload.

Hand hygiene prevents healthcare-associated infections,^1^ but proper hand hygiene takes time, which is limited as workload increases. Pittet et al^2^ found an inverse relationship between hand hygiene compliance and workload, measured by the number of hand hygiene opportunities per hour, which suggests that healthcare workers may sacrifice hand hygiene compliance as workload increases. Voss and Widmer^3^ estimated that 12 healthcare workers would need 4 hours to do hand hygiene with an alcohol hand rub during an 8-hour shift in an intensive care unit if they were 100% compliant. Given their estimates, they asked the provocative question, “Is 100% compliance with hand-cleansing routines attainable, and, if so, can we afford it?”^3^ Haac et al^4^ observed an average of 34 hand hygiene opportunities per trauma resuscitation and documented 7% compliance with the WHO Hand Hygiene Moments and 0% compliance before clean procedures. They also questioned whether 100% compliance is attainable in such settings. In our literature search, we did not find multicenter studies that assessed hand hygiene compliance at different workload levels. Thus, we conducted a retrospective analysis to address this gap and to determine whether limits exist for hand hygiene compliance in relation to workload.

## Methods

We calculated hand hygiene compliance rates for healthcare workers observed during the STAR*ICU study (see Supplementary Appendix online).^5^ Healthcare workers were considered compliant if they used alcohol-based hand rubs or cleaned their hands with soap and water. We defined hand hygiene opportunities as the transitions between tasks and workload as the total number of hand hygiene opportunities during a single patient observation session.^2^ We assessed workload as a continuous variable and we also categorized it into tertiles: low (≤ 12 opportunities per hour); medium (13–20 opportunities per hour), and high (>20 opportunities per hour).

We used χ^2^ tests for the difference between proportions to determine whether hand hygiene compliance varied significantly between workload categories. We used logistic regression, adjusting for healthcare worker type, glove use, and the presence of isolation precautions, to assess the effect of workload on hand hygiene compliance. We performed separate logistic regression analyses for continuous and categorical workload data. We used SAS version 9.4 software (SAS Institute, Cary, NC) for the statistical analyses.

## Results

**Fig. 1.**
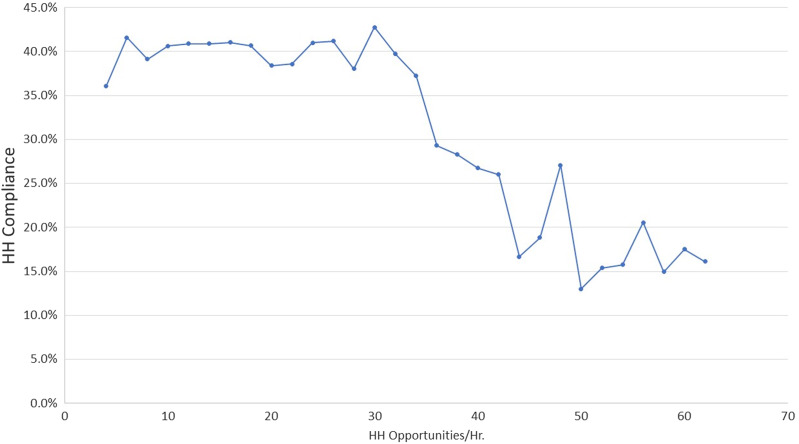
Hand hygiene (HH) compliance by the number of HH opportunities per hour.

We identified 42,349 hand hygiene opportunities in the STAR*ICU dataset. As workload increased, the proportion of hand hygiene opportunities associated with nurses, with glove use, and with care provided to patients in isolation precautions decreased significantly (Table 1). The association between the continuous workload variable and hand hygiene compliance remained stable until the workload approached 30 hand hygiene opportunities per hour, after which compliance decreased significantly (Fig. 1). The probability model, which evaluated the data as a continuous variable, predicted a ∼1% reduction in observed compliance for every additional opportunity per hour (OR, 0.99; 95% CI, 0.99–0.99; *P* < .0001).

**Table 1.** Observed Hand Hygiene Compliance by Workload Level

Variable	Low Workload				Medium Workload				High Workload			
		HH Compliance				HH Compliance				HH Compliance		
	No. (%)	%	95% CI		No. (%)	%	95% CI		No. (%)	%	95% CI	
Overall	14,832	39.9	38.7	40.5	13,833	40.4	39.1	41.0	13,684	36.7	35.3	37.3
**Healthcare worker type**												
Nurse	11,816 (79.7)	42.7	41.8	43.2	9,580 (69.3)^a^	44.3	43.3	44.8	8,182 (59.8)^a^	41.4	40.3	41.9
Physician	10,65 (7.2)	28.0	25.3	29.3	1,825 (13.2)^a^	33.3	31.1	34.3	2,880 (21.0)^a^	30.5	28.8	31.3
Other	1,951 (13.2)	29.4	27.4	30.4	2,428 (17.6)^a^	30.4	28.6	31.3	2,622 (19.2)^a^	28.8	27.0	29.6
**Glove use**												
Yes	6,368 (42.9)	51.6	50.4	52.2	6,270 (45.3)^a^	50.4	49.1	51.0	5,751 (42.0)	50.4	49.1	51.1
No	8,464 (57.1)	31.1	30.1	31.6	7,563 (54.7)^a^	32.1	31.1	32.6	7,933 (58.0)	26.7	25.7	27.2
**Presence of isolation**												
Yes	3,357 (22.6)	44.5	42.8	45.3	29,61 (21.4)^a^	42.5	40.7	43.4	1,964 (14.4)^a^	36.9	34.7	37.9
No	11,475 (77.4)	38.9	38.0	39.3	10,872 (78.6)^a^	39.8	38.9	40.3	11,720 (85.6)^a^	36.6	35.8	37.1
**Odds of hand hygiene compared with low workload^b^**												
					OR	95% CI		*P* Value	OR	95% CI		*P* Value
Unadjusted	Ref				1.02	0.97	1.07	.41	0.87	0.83	0.91	<.0001
Adjusted	Ref				0.98	0.93	1.03	.14	0.93	0.89	0.98	<.0001

Note. N, number of hand hygiene opportunities; HH, hand hygiene; OR, odds ratio; CI, confidence interval.

a *P* < .05 when comparing the proportion of hand hygiene opportunities associated with each subgroup (healthcare worker type, glove use, presence of isolation) at medium and at high workloads with that at low workloads.

b Unadjusted and adjusted for healthcare worker type, glove use, and presence of isolation.

Hand hygiene compliance did not differ significantly between low and medium workload periods, but compliance was significantly lower during high workload periods than during low workload periods (Table 1). The unadjusted odds for compliance did not differ significantly between low and medium workload periods, but the odds decreased significantly from low to high workload periods (OR, 0.87; 95% CI, 0.83–0.91; *P* < .0001). After adjusting for healthcare worker type, glove use, and isolation precautions, the difference in the odds of compliance between low and high workload periods remained significant, but the magnitude of the difference decreased (OR, 0.93; 95% CI, 0.89–0.98; *P* < .0001) (Table 1).

As the workload level increased, hand hygiene compliance rates of nurses and other healthcare workers did not change significantly, but those of physicians increased, especially between low and medium workloads. However, their compliance remained lower than that of nurses. Compliance during opportunities associated with isolation precautions decreased significantly at high workload compared with low and medium workloads. Moreover, as workload increased, the number of unique healthcare worker types and the proportion of sterile and open-wound–related tasks during an observation period also increased (Supplementary Tables 1 and 2).

## Discussion

Hand hygiene compliance was ∼40% until the workload reached 30 opportunities per hour, at which point compliance decreased by ∼1% per additional hand hygiene opportunity per hour, which was greater than that reported by Pittet et al.^2^ This finding suggests that healthcare workers cannot incorporate an unlimited number of hand hygiene opportunities into their patient care processes and still perform the necessary tasks. Thus, interventions to improve hand hygiene compliance may not be effective if the total number of opportunities is above this limit.

The percentage of hand hygiene opportunities performed by physicians and other healthcare workers increased significantly as workload increased, which may account somewhat for the decrease in overall hand hygiene compliance given that their compliance was significantly worse than that of nurses at all workload levels. Other groups have also found lower compliance among physicians than among nurses.^6^ In our study, compliance associated with isolation precautions decreased with increased workload. In a study by Dhar et al,^7^ as the proportion of patients in isolation precautions increased, compliance with both isolation protocols and hand hygiene decreased significantly.^7^ In our study, the number of different healthcare worker types increased as workload increased, as did the proportion of tasks involving open wounds and sterile procedures, which may indicate that the complexity of patient care increased and that the possibility of contaminating sterile sites might increase with workload. Complex patient care tasks may take more time than other tasks and, thereby, reduce the time available for hand hygiene. The results of our study and the studies by Dhar et al^7^ and by Haac et al^4^ suggest that healthcare workers may sacrifice hand hygiene compliance to complete patient care tasks. Our results also support the contention Voss and Widmer that healthcare workers may be unable to reach 100% hand hygiene compliance, particularly during periods of high-intensity patient care.^4^

Our study is unique in that we included multiple centers and we stratified our analyses of hand hygiene compliance by workload level. The STAR*ICU study was conducted in 2005–2006 and hand hygiene compliance likely has improved since then. However, this trend is likely to shift “compliance by opportunities curve” upward rather than nullifying our results. This interpretation is supported by the difference between our curve and that found by Pittet et al. In their study, which was conducted in 1994, compliance dropped starting at 10 opportunities per hour and decreased 5% ± 2% for every 10 additional opportunities thereafter. We found a ∼1% decrease for every additional opportunity over the whole curve, with a steep compliance decline after 30 opportunities per hour.

In this study, hand hygiene compliance remained stable between 5 and 30 hand hygiene opportunities per hour and decreased dramatically when the number of opportunities exceeded 30 per hour. This result suggests that healthcare workers can integrate only a limited number of hand hygiene opportunities into their patient care processes. In addition, the proportion of hand hygiene opportunities for which physicians were responsible increased at higher workload levels, physicians’ hand hygiene compliance was low in general, and compliance for opportunities associated with isolation precautions was very low. These 3 factors accounted for much of the observed reduction. Thus, future studies of the association between workload and hand hygiene compliance should evaluate how such factors interact with workload to affect compliance. Our results suggest that hand hygiene interventions designed to improve compliance before critical procedures (eg, accessing indwelling devices),^8^ compliance by physicians, and compliance during care for patients in isolation may be more effective than interventions targeting all hand hygiene opportunities. In addition, healthcare facilities should provide adequate staffing during higher workload periods to ensure that providers maintain a high level of hand hygiene compliance.

## Supplementary material

For supplementary material accompanying this paper visit https://doi.org/10.1017/ice.2021.179. click here to view supplementary material
