# Supplementary material for: The impact of workload on hand hygiene compliance: Is 100% compliance achievable?
Source: Infect Control Hosp Epidemiol. 2021 May 14;43(9):1259–61. doi: 10.1017/ice.2021.179 (PMC9483712; doi:10.1017/ice.2021.179)
Supplement: Supplementary file 1 [file S0899823X21001793sup001.docx]

Supplementary Material:

**Appendix I.** Data Collection for Hand Hygiene Compliance:

During the STAR*ICU study, trained monitors observed patient care in individual patients’ rooms for 30 minute periods. They collected information about healthcare providers’ contacts with the patients, the patients’ devices, and the patients’ environment. For the STAR*ICU study, hand hygiene opportunities were defined as the transitions between task. Monitors assessed whether healthcare providers did hand hygiene after the first task and before the second task. If healthcare workers performed hand hygiene using soap and water or alcohol-based hand rubs, they were considered compliant for the transition/opportunity.

Contact 1

Contact 2

HH Before

HH After

HH After

HH Before

Transition

Hand Hygiene Opportunity

**Appendix II:** Supplementary Table 1:Task Type Distribution by Workload

|  | Low | | Medium | | High | | Total |
| --- | --- | --- | --- | --- | --- | --- | --- |
| Task Type | N | % | N | % | N | % | N |
| Contaminated-Eliminations | 408 | 28.7% | 522 | 36.7%* | 492 | 34.6% | 1422 |
| Environment | 3601 | 33.0% | 3401 | 31.2% | 3896 | 35.7%* | 10898 |
| Patient | 2596 | 31.5% | 2796 | 33.9% | 2861 | 34.7%* | 8253 |
| Contaminated-Open Wound | 78 | 19.4% | 138 | 34.3%* | 186 | 46.3%* | 402 |
| Contaminated-Respiratory | 680 | 31.5% | 765 | 35.4% | 714 | 33.1% | 2159 |
| Contaminated-Urinary | 234 | 33.8% | 218 | 31.5% | 240 | 34.7% | 692 |
| Device-Other | 772 | 32.0% | 784 | 32.5% | 854 | 35.4% | 2410 |
| Device-Blood or Bodily Fluids | 511 | 33.9% | 494 | 32.8% | 503 | 33.4% | 1508 |
| Blood | 302 | 31.9% | 331 | 35.0% | 313 | 33.1% | 946 |
| Sterile | 15 | 11.0% | 35 | 25.7% | 86 | 63.2%* | 136 |

* p < 0.05 when comparing the proportion of hand hygiene opportunities associated with each subgroup at medium and at high workloads with that at low workloads.

**Appendix III:** Supplementary Table 2: Number of Unique Healthcare Worker Types during Single Observation Periods by Workload

|  | Number of Observation Periods (N = 5635; %) | Number of Observation Periods (N = 4349; %) | | | | Number of Observation Periods (N = 3539; %) | | |
| --- | --- | --- | --- | --- | --- | --- | --- | --- |
| 1 | 3827 (69.9%) | 1695 (40.0%) | | | | 970 (27.4%) | |  |
| 2 | 1675 (29.7%) | 1598 (36.7%) | |  |  | 958 (27.1%) |  |  |
| 3 | 133 (2.4%) | 883 (20.4%) | |  |  | 826 (23.3%) | | |
| ≥4 | 0 (0%) | 168 (3.9%) |  |  |  | 785 (22.2%) |  |  |
